# Injury patterns and causes of death in 953 patients with penetrating abdominal war wounds in a civilian independent non-governmental organization hospital in Lashkargah, Afghanistan

**DOI:** 10.1186/s13017-019-0272-z

**Published:** 2019-11-21

**Authors:** Maurizio Cardi, Khushal Ibrahim, Shah Wali Alizai, Hamayoun Mohammad, Marco Garatti, Antonio Rainone, Francesco Di Marzo, Giuseppe La Torre, Michela Paschetto, Ludovica Carbonari, Valentina Mingarelli, Andrea Mingoli, Giuseppe S. Sica, Simone Sibio

**Affiliations:** 1 Emergency NGO Medical Division, Lashkargah Hospital, Lashkargah, Afghanistan; 2 Dipartimento di Chirurgia “P. Valdoni”, Sapienza Università di Roma, Viale del Policlinico155, 00161 Rome, Italy; 3 Chirurgia Generale, Fondazione Poliambulanza, Istituto Ospedaliero, Via Bissolati 57, Brescia, Italy; 4 Chirurgia Generale, Ospedale Versilia, Via Aurelia 335, 55041 Lido di Camaiore, LU Italy; 5 Dipartimento di Sanità Pubblica e Malattie Infettive, Sapienza Università di Roma, Viale del Policlinico155, 00161 Rome, Italy; 6 Dipartimento di Chirurgia, Università Tor Vergata, Viale Oxford 81, 00133 Rome, Italy

**Keywords:** Penetrating abdominal injuries, Abdominal war wounds, War damage control, Gastrointestinal war injuries

## Abstract

**Background:**

Management of penetrating abdominal war injuries centers upon triage, echeloned care, and damage control. A civilian hospital based in a war zone can rarely rely upon these principles because it normally has limited resources and lacks rapid medical evacuation. We designed this study to describe organ injury patterns and factors related to mortality in patients with penetrating abdominal war injuries in a civilian hospital in an active war zone in Afghanistan, examine how these findings differ from those in a typical military setting, and evaluate how they might improve patients’ care.

**Methods:**

We reviewed the records of all patients admitted at the Lashkargah “Emergency” hospital with penetrating abdominal injuries treated from January 2006 to December 2016. Demographic and clinical data were recorded; univariate and multivariate analyses were used to identify variables significantly associated with death.

**Results:**

We treated 953 patients for penetrating abdominal injury. The population was mainly civilian (12.1% women and 21% under 14). Mean age was 23 years, and patients with blast injuries were younger than in the other groups. The mechanism of injury was bullet injury in 589 patients, shell injury in 246, stab wound in 97, and mine injury in 21. The most frequent abdominal lesion was small bowel injury (46.3%). Small and large bowel injuries were the most frequent in the blast groups, stomach injury in stab wounds. Overall mortality was 12.8%. Variables significantly associated with death were age > 34 years, mine and bullet injury, length of stay, time since injury > 5 h, injury severity score > 17, and associated injuries.

**Conclusions:**

Epidemiology and patterns of injury in a civilian hospital differ from those reported in a typical military setting. Our population is mainly civilian with a significant number of women and patients under 14 years. BI are more frequent than blast injuries, and gastrointestinal injuries are more common than injuries to solid organs. In this austere setting, surgeons need to acquire a wide range of skills from multiple surgical specialties. These findings might guide trauma and general surgeons treating penetrating abdominal war wounds to achieve better care and outcome.

## Background

From 7 to 17% of all war casualties involve the abdomen [1–7], and abdominal organs are particularly vulnerable to penetrating trauma characterized by multiple injuries to the gastrointestinal tract, solid organs, and vascular structures, given that bullets and shells have high kinetic energy [8–10]. Although overall mortality rates for war wounds decreased from more than 30% during World War II to less than 10% in recent conflicts [9], penetrating abdominal injuries are a major cause of preventable deaths and still carry significant morbidity and mortality.

Today’s surgical strategy in the management of penetrating abdominal war injuries centers upon triage, echeloned care, and damage control, followed by rapid evacuation to a referral hospital for definitive treatment [11], but with the exception of reports from the International Committee of the Red Cross (ICRC) field hospitals [10, 12–14], nearly all reports come from military hospitals or civilian referral hospitals treating the war wounded [2–4, 7, 8, 15–18]. For many reasons, a civilian hospital based in a war zone can rarely rely upon these principles because it normally has limited resources, lacks echeloned care and rapid medical evacuation, and has to undertake primary as well as secondary surgery. Equally important, epidemiology presumably differs in civilian and military settings because a civilian population is typically unprotected from body armors and has a more variable pattern of injuries [19] and lengthy delay from injury to hospital treatment. Having a better insight into these possible differences might improve these patients’ care and outcome.

We designed this study primarily to describe organ injury patterns and factors related to mortality in patients with penetrating abdominal war injuries in a civilian hospital in an active war zone in Afghanistan. We then examined how these findings differ from those amply reported from a typical military setting or civilian referral hospitals treating war wounded. Finally, we evaluated how this previously unavailable information might improve patients’ care despite the limited resources in an austere environment.

## Methods

We retrospectively reviewed the records of all patients with penetrating abdominal injuries admitted and operated upon at the “Emergency” non-governmental (NGO) hospital in Lashkargah, Afghanistan, from January 2006 to December 2016. Patients already treated elsewhere or transferred from other hospitals were excluded from the study. The hospital is an 80-bed facility built by Emergency in 2004 and is a limited resources hospital. It has no conventional intensive care unit, only basic radiologic equipment, no computerized tomographic scanning, and can only do routine laboratory tests.

All patients were initially assessed upon arrival in the emergency room with a rapid primary survey following the Advanced Trauma and Life Support (ATLS) guidelines [20] and specific “Emergency” guidelines for the management of abdominal trauma. In patients arriving in shock (systemic blood pressure < 90 mmHg, heart rate > 100 bpm), the Airway, Breathing, Circulation, Disability, Exposure (ABCDE) priorities protocol was often replaced by C-AB. All patients received supplemental oxygen (8 l/min by valve/reservoir device with the reservoir bag inflated), and a nasogastric tube and Foley catheter were placed unless contraindicated. Chest and pelvic X-rays were done on clinical suspicion only after life-threatening injuries had been recognized and treated. Adequate intravascular volume was restored by inserting two large-bore peripheral cannulas in the antecubital veins of the arms. If needed, other peripheral (external jugular, femoral) lines, venous cutdowns (saphenous vein at the foot), or central venous line were used, according to the patient’s needs and staff skills. Blood was drawn for type and crossmatch and baseline laboratory tests (hemoglobin, hematocrit, arterial blood gases), and crystalloid infusion with ringer lactate or normal saline or both was then started. All patients received standard antibiotic therapy (ampicillin 1 g iv QID for 24 h or until they resumed oral diet, followed by 500 mg per os QID for the next 4 days, and chloramphenicol, 1 g iv QID for 24 h or until they resumed oral diet, followed by 500 mg per os QID for the next 4 days). Metronidazole 500 mg iv TID until NPO followed by 500 mg per os QID up to 5 days was added if a colon injury was found at operation.

Indications for laparotomy were obvious penetrating injury to the abdomen, clinically tender abdomen with signs of peritonitis, patients hemodynamically unstable or in shock on admission or during another surgical procedure, bowel evisceration, and obvious bleeding from the stomach, rectum, or genitourinary tract. Patients with uncontrollable abdominal bleeding and multiple intra-abdominal or associated extraabdominal injuries, or both requiring long surgical procedures underwent damage control laparotomy (DCL). Laparotomy included intra-abdominal packing for bleeding control, small or large bowel resections, or both without anastomoses and temporary abdominal closure (Bogota bag). Relaparotomy was scheduled 24–48 h later for removal of packing, definitive treatment of the abdominal injuries, intestinal anastomosis, and abdominal closure.

Data were retrieved from a prospectively collected electronic database regularly updated by the international medical personnel. Demographic data were collected, such as age, gender, mechanism of injury, and time since injury. According to the mechanism of injury, patients were divided into four groups: bullet injury (BI), shell injury (SI), mine injury (MI), and stab wounds (SW). Other variables recorded were number of negative laparotomies, defined as a laparotomy in patients without intra- or retroperitoneal injuries, site of injury, surgical procedures, length of hospital stay, and complication and mortality rates. Severity of the injury was assessed using the injury severity score (ISS) [21]. Associated injuries were also recorded and divided into six categories: head and neck, chest, extremities, pelvis, peripheral vascular, and spinal column.

### Statistical analysis

Data are presented as frequency distribution and contingency tables. Differences between groups were assessed by non-parametric tests (*χ*^2^ and Kruskal-Wallis for qualitative, Mann-Whitney for quantitative tests). In the multivariate analysis, receiver operating characteristic (ROC) curves for the outcome “death” were used to establish cutoff points for time since injury, ISS, and age data. A multiple logistic regression model was built to verify the variables significantly associated with death. Results were shown as odds ratio (OR) and 95% confidence intervals using a stepwise approach (backward elimination). *P* values less than 0.05 were considered to indicate statistical significance. Statistical data were analyzed with SPSS (Statistical Package for Social Sciences, v. 25, 2017. Chicago, IL).

## Results

From January 2006 to December 2016, 1095 patients were admitted and treated for penetrating abdominal war injuries. We excluded from the study 131 patients already treated in other hospitals or with incomplete records and 11 patients with a negative laparotomy, leaving 953 patients for evaluation. Women accounted for an overall 12% incidence but with a higher incidence in the SI group (Table 1). The mechanism of injury was a BI in 589 patients (61.8%), an SI in 246 (25.8%), an SW in 97 (10.1%), and an MI in 21 (2.2%). The study population was young (mean age 22.4 years), 12.3% of the patients were under 10, and 41% under age 20. Patients with SI were significantly younger than those in the other groups: 25.2% of the patients were younger than 10 and 57.7% younger than 20 years (Table 2).

**Table 1:** Demographics and clinical characteristics. Data are expressed as median (interquartile range)

Characteristics	Total (*n* = 953)	BI (*n* = 589)	SI (*n* = 246)	SW (*n* = 97)	MI (*n* = 21)	*P* values
Age (years)	23 (17–30)	25 (20–30)	19 (10–28)	25 (20–28)	21 (15–25)	< 0.001^^^
Men (*N*, %)	838 (87.9)	524 (89)	204 (82.9)	91 (93.8)	19 (90.5)	0.021^§^
Injury severity score	16 (9–25)	16 (16–25)	22 (16–39)	9 (9–18)	18 (16–25)	< 0.001^^^
Length of stay	8 (6–12)	8 (6–13)	8 (6–12)	6 (5–7)	8 (3–11)	< 0.001^^^
Time since injury°	5.4	5.1	4.4	6.2	8.2	0.005^^^
Associated injuries (*n*, %)	399 (41.8)	242 (41)	117 (47.5)	29 (29.8)	12 (57.1)	< 0.005^§^

*Data expressed as *N* (percent)

^°^Data expressed as mean (hours)

^^^Kruskal-Wallis test

^§^*χ*^2^ test

**Table 2:** Age distribution and mechanism of injury. Data expressed as number (%)

Mechanism of injury	Age groups (years)				
	1–10	11–20	21–40	41–60	> 60
Bullet injury (*n* = 589)	46 (7.8)	155 (26.3)	322 (54.7)	56 (9.5)	10 (1.7)
Shell injury (*n* = 246)	62 (25.2)	80 (32.5)	73 (29.7)	18 (7.3)	13 (5.3)
Stab wound (*n* = 97)	7 (7.2)	31 (31.9)	46 (47.5)	11 (11.3)	2 (2.1)
Mine injury (*n* = 21)	2 (9.5)	8 (38.1)	10 (47.6)	1 (4.8)	–
Total patients (*n* = 953)	117 (12.3)	274 (28.7)	451 (47.4)	86 (9)	25 (2.6)

*χ*^2^ 82.109

*p* <  0.00001

The mean ISS score was 18.7, significantly higher in patients with SI than in the other groups (Table 1). Of the 953 patients studied, 399 patients (41.9%) reported associated injuries, more frequent in patients with blast injuries (47.5% in the SI and 57.1% in the MI groups). Injuries to multiple body regions (≥ 2) were found in 10.1% of the patients but were higher in patients with blast injuries (25.2% in SI and 23.8% in MI). A mean 5.4 h elapsed between injury and hospital admission. Patients with blast injuries arrived significantly later in hospital than patients with other injuries (MI 8.2 and SI 6.2 h, *p* <  00.5) (Table 1).

Of the 953 patients treated, 43 (4.5%) underwent a DCL. Eleven of these 43 patients died of hemorrhagic shock or severe associated extra-abdominal injuries before relaparotomy for the definitive treatment of abdominal injuries and primary abdominal closure. Five died (2 of associated severe brain injury and 3 of sepsis) and 27 survived (62.8%).

The most common abdominal lesions were gastrointestinal injuries (small and large bowel, stomach, duodenum, and rectum) (Table 3). The small bowel was the single most frequent intra-abdominal injury (46.3%), followed by the colon (37.8%), liver (16.7%), stomach (10.9%), diaphragm (10.9%), and kidney (6.8%). Small and large bowel injuries were more frequent in the blast groups (MI and SI) than in the other groups, whereas stomach injury was more frequent in stabbed patients than in the other groups (23.7% vs. 13.9% in BI, 13% in SI, and 4.8% in MI groups). Injuries to solid organs (spleen, liver, kidneys, and pancreas) were less common and more frequent in patients with BI than in the other groups. Multiple segment intestinal injuries in the small and large bowel were found in 21.3% of the patients, with no difference between the 4 groups. The single most common operation in the 953 patients was small bowel resection and anastomosis. Of the 953 patients, 495 (49.1%) needed a resection or anastomosis to repair bowel injuries, whereas 488 (50.4%) had a primary suture (Table 4).

**Table 3:** Distribution of abdominal injuries according to the mechanism of injury. Data are expressed as frequency (%)

Site of injury	*N*	Mechanism of injury				*P* values
		Bullet injury (*n* = 589)	SI (*n* = 246)	SW (*n* = 97)	MI (*n* = 21)	
Small bowel	441	267 (45.3)	128 (52)	31 (31.9)	15 (71.4)	< 0.0001
Colon	290	240 (40.7)	86 (30.9)	20 (20.6)	9 (42.8)	0.0015
Liver	160	107 (18.2)	40 (16.3)	13 (13.4)	3 (14.3)	NS
Stomach	104	82 (13.9)	32 (13)	23 (23.7)	1 (4.8)	< 0.05
Kidney	45	50 (8.5)	9 (3.6)	3 (3.1)	1 (4.8)	< 0.05
Spleen	55	38 (6.4)	12 (4.9)	4 (4.1)	1 (4.8)	NS
Diaphragm	100	70 (11.9)	19 (7.7)	9 (9.3)	–	NS
Bladder	37	31 (5.3)	5 (2)	–	–	< 0.05
Rectum	55	55 (9.3)	8 (3.2)	–	1 (4.8)	< 0.0005
Duodenum	34	20 (3.4)	9 (3.6)	5 (5.1)	–	NS

*χ^2^ test

*NS* not significant

**Table 4:** Technique of intestinal repair according to the site of injury

Site of injury	*n*	Technique of intestinal repair	
		Resection/anastomoses, *n* (%)	Primary suture, *n* (%)
Small bowel	444	271 (61)	173 (39)
Large bowel	362	184 (50.8)	178 (49.2)
Stomach	104	2 (2)	102 (98)
Rectum	55	36 (65.5)	19 (34.5)
Duodenum	34	–	34 (100)
Total	999	493 (49.3)	506 (50.7)

The overall complication rate was 38.9% with no significant differences between the 4 groups (Table 5). The most common complications were lung-related (pneumonia and acute respiratory distress syndrome, 12.9%). An anastomotic leak developed in 13 patients (2.8%) with a higher incidence for colonic than for small bowel anastomoses (3 of 271, 1.1% versus 10 of 184, 5.4%).

**Table 5:** Postoperative complications

Complication	*N* (%)
Lung-related	123 (12.9)
Other extra-abdominal infections	84 (8.8)
Intra-abdominal infections	76 (7.9)
Wound infections	57 (5.9)
Anastomotic leak	13 (2.8) *
Intraabdominal hemorrhage	18 (1.8)
Death	122 (12.8)
Total	375 (38.9)

*Related to the number of anastomoses (*n* = 464)

Of the 953 patients treated, 122 (12.8%) died from the injuries sustained. The mortality rate was higher in patients with stab wounds and mine injuries than in the other groups (Table 6). The most frequent cause of death was hemorrhagic shock (73.7%) with no significant differences between groups (Table 7). Univariate analysis showed that associated chest and peripheral vascular, right and transverse colon, right diaphragm, right kidney, duodenum, inferior vena cava (IVC), pancreas, iliac artery, portal vein, and superior mesenteric artery injuries were significantly associated with higher mortality rates (Table 8). Multivariate analysis identified the clinical variables linked to death as age > 34, MI and BI, length of stay, time since injury > 5 h, ISS > 17, and presence of associated injuries (Table 9).

**Table 6:** Association between mechanism of injury and death

Mechanism of injury	Alive		Died		χ^2^	*P*
	*N*	%	*N*	%		
Bullet (*n* = 589)	502	85.2	87	14.8	19.39	< 0.001
Shell (*n* = 246)	217	88.5	28	11.4		
Stab (*n* = 97)	97	98.9	1	1.1		
Mine (*n* = 21)	15	71.4	6	28.6		
Patients (*n* = 953)	831	87.2	122	12.8

**Table 7:** Causes of death according to the mechanism of injury

Variables	Entire cohort (*n* = 952)	Bullet (*n* = 589)	Shell (*n* = 245)	Stab (*n* = 97)	Mine (*n* = 21)	*P* values
Multi organ failure, *n* (%)	7 (0.7)	6 (1.0)	1 (0.4)	0 (–)	0 (–)	0.6
Sepsis, *n* (%)	10 (1.1)	8 (1.4)	1 (0.4)	0 (–)	1 (4.8)	0.2
Hemorrhage, *n* (%)	90 (9.5)	66 (11.2)	21 (8.6)	1 (1.0)	2 (9.5)	0.02
Brain injury, *n* (%)	5 (0.5)	3 (0.5)	2 (0.8)	0 (–)	0 (–)	0.8
Other, *n* (%)	10 (1.1)	5 (0.8)	3 (1.2)	0 (–)	2 (9.5)	0.001
Total, *n* (%)	122 (12.8)	88 (14.9)	28 (11.4)	1 (1.0)	5 (23.8)	0.001

**Table 8:** Association between site of injury and death

Site of injury	Patients wounded		Alive		Died		*χ*^2^	*P*
	*N*	%	*N*	%	*N*	%		
Small bowel	441	46.3	383	86.9	58	13.1	0.090	0.764
Left colon	148	15.5	131	88.5	17	11.5	0.271	0.602
Right colon	142	14.9	110	77.5	32	22.5	14.273	< 0.001*
Right liver	140	14.7	116	82.8	24	17.2	2.751	0.097
Stomach	104	10.9	89	85.5	15	14.5	0.275	0.600
Transverse colon	79	8.3	58	73.4	21	26.6	14.654	< 0.001*
Right diaphragm	56	5.9	43	76.8	13	23.2	5.779	0.016*
Spleen	55	5.8	45	81.8	10	18.2	1.514	0.219
Rectum	55	5.8	45	81.8	10	18.2	1.514	0.219
Left diaphragm	44	4.6	39	88.6	5	11.4	0.085	0.770
Right kidney	39	4.1	30	76.9	9	23.1	3.846	0.050*
Bladder	37	3.9	28	75.7	9	24.3	4.579	0.032*
Duodenum	34	3.6	23	67.6	11	32.4	12.047	0.001*
Left kidney	26	2.7	23	88.5	3	11.5	0.038	0.845
Gallbladder	20	2.1	16	80	4	20	0.948	0.330
Left liver	20	2.1	15	75	5	25	2.723	0.099
Inferior vena cava	17	1.8	8	47	9	53	24.982	< 0.001*
Iliac vein	14	1.5	11	78.5	3	21.5	0.947	0.330
Iliac artery	12	1.3	8	66.6	4	33.4	4.589	0.035*
Pancreas	10	1	6	60	4	40	6.697	0.010*
Left ureter	9	0.9	7	77.7	2	22.3	0.722	0.395
Retroperitoneal hematoma	8	0.8	6	75	2	25	1.075	0.300
Aorta	6	0.6	5	83.3	1	16.7	0.081	0.776
Right ureter	5	0.5	4	80	1	20	0.233	0.629
Portal vein	5	0.5	2	40	3	60	10.031	0.002*
Uterus	2	0.2	2	100	–	–	0.294	0.588
Heart	2	0.2	1	50	1	50	2.484	0.115
Vagina	1	0.1	1	100	–	–	0.147	0.701
Ovary	1	0.1	1	100	–	–	0.147	0.701
Mesenteric artery	1	0.1	–	–	1	100	6.819	0.009*

**Table 9:** Multivariate analysis of variables associated with death

Variable	Odd ratio	95% confidence interval
Age > 34	2.45	1.54–3.91
Mine injury	5.69	1.81–17.82
Bullet injury	1.98	1.21–3.21
Length of hospital stay	0.97	0.95–0.99
Inferior vena cava injury	4.72	1.53–14.59
Bladder injury	3.31	1.32–8.31
Duodenum injury	2.61	1.06–6.41
Transverse colon injury	4.01	2.19–7.35
Right colon injury	2.86	1.71–4.79
Portal vein injury	8.62	1.13–65.50
Associated injuries (Y/N)	3.40	2.19–5.28
Time since injury > 5 h	1.86	1.20–2.88
Injury severity score > 17	6.92	16.29–97.1

## Discussion

In this study, we provide previously unavailable information on epidemiology, organ injury patterns, and factors related to mortality in patients with penetrating abdominal war injuries in a limited resources civilian hospital in an active war zone in Afghanistan. These findings seem to differ from reported information referring to typical military settings. Our population is young, includes many women, and has a long delay between injury and treatment. Knowing more about the differences between the two hospital settings should help to improve the way we care for these patients and manage their injuries.

Our patients were all admitted to the hospital of an international, independent NGO (“Emergency”) founded in 1994 to provide free-of-charge surgical and medical assistance to war victims. In the civilian hospital of Lashkargah, the capital city of the Helmand province, in the epicenter of the Afghan conflict, no medical evacuation is possible and the same hospital is responsible for primary and secondary surgery. Surgeons therefore face the difficult professional challenge to deal with a wide range of procedures including war wound management, orthopedic and vascular surgery, craniotomies, and at least basic plastic reconstructive surgery.

In this environment, we found that epidemiology and organ injury patterns significantly differ from typical military or civilian referral hospitals treating war-wounded patients, influencing care for the victims and clinical outcome. Unlike previous studies describing patients treated in military hospitals, our study population was mainly civilian with a significant number of women and patients under 14 years and, equally important, more than 5 h delay between injury and hospital treatment. Access to care for the civilian population in Afghanistan, and especially in Helmand province, is difficult owing to the lack of health facilities, geographic landscape, mined areas, and ongoing armed conflict. To facilitate the referral and treatment of patients wounded in remote areas far from the main hospitals, “Emergency” has established and gradually expanded a unique widespread network of first aid posts (FAP), where local staff, trained in the “Emergency” hospitals, can provide basic healthcare and first aid and stabilize wounded patients, referring those who need definitive surgical treatment to the main hospitals through an ambulance service available 365 days a year. In the past years, the 47 FAPs in 10 provinces referred more than 30% of hospital admissions, reporting a fatality rate of less than 1% during transportation [22].

When we investigated organ injury patterns, contrary to other studies from military or civilian hospitals treating war or terrorist’s attack injuries [23, 24], BI were significantly more frequent than blast injuries. In most military papers, the incidence of blast wounds is higher than BI, as observed by Arafat [25] during the Syrian war (56 vs. 43%), Stevenson [15] in Iraq-Afghanistan (56 vs. 24%), and Pasquier [3] in Iraq-Afghanistan (73 vs. 27%). By far, the most frequent intra-abdominal injury observed was the small bowel, followed by colon injuries. Solid organs (liver, spleen, and kidney) were involved less frequently, but had a higher incidence in the BI than in the blast group (33 vs. 24%). This difference probably arises because solid organs are partially protected by boney structures and need higher energy for damage. Kinetic energy depends more on velocity than mass (*E* = *mv*^2^), and fragments generated by explosions normally travel at a lower speed than a bullet (approximately 400 vs. 900–1000 m/sec). The optimal management of gastrointestinal tract war injuries is still controversial. Bowel injuries can range from full-thickness disruption to mural hematoma with various degrees of submucosal hemorrhage, and the optimal management should take into consideration number, location, size, and type of the injury [26, 27]. In our experience, for managing multiple, jejunal, and large (> 50% wall circumference) injuries, we usually prefer a resection rather than a primary repair, with a low incidence of anastomotic leaks (2.8%), higher for colonic than for small bowel anastomoses (5.4 vs. 1.1%).

A controversial surgical decision in managing colorectal injuries regards the need for a protective colostomy. For many surgeons, a repair without a proximal stoma is the main factor leading to leakage and peritonitis, and a colostomy should always be used as in rectal lesions [4, 28, 29], whereas for others it is safe [30]. In our group of patients, we did a colostomy in only 13.6% of the patients with colorectal injury, almost all owing to gross fecal contamination or during DCS.

Another management concern in war settings is DCL. In our group of patients, we used a DCL in few cases (43, 4.5%) with a 62.7% survival rate. Although abbreviated laparotomy can be used to control diffuse bleeding and gross contamination, the limited availability of blood products to correct blood loss and coagulation factor deficiency and lack of sophisticated hemodynamic monitoring systems in an austere environment could fail to reverse hypothermia, coagulation disorders, and acidosis [31]. In their study on penetrating abdominal trauma following terrorist bombing attacks, Bala et al. used DCL in 4 patients and reported 50% of survival [25]. Similarly, Fries et al. [32] in deployed UK military personnel used DCL in 7/22 patients with abdominal trauma from both gunshot and blast wounds reporting no mortality, concluding that military surgeons may underuse DCL. In the US deployed military during OEF and OIF, Mitchell et al. [33] reported 331 DCLs achieving the same mortality rates as definitive laparotomy (1.5 vs. 1.4%). In a Medline review of abdominal blast injuries including civilian and military reports, Turégano-Fuentes concluded that DCL should be the rule in unstable patients, particularly owing to the high risk of abdominal compartment syndrome and therefore recommended using a temporary abdominal closure [26]. Our findings seem to show that despite limited resources and the related problems, DCL should be considered for managing patients with complex combined visceral injuries.

A major feature we studied were factors related to mortality. As expected in patients with penetrating abdominal injuries, the most frequent cause of death was hemorrhagic shock (73.7%) with no significant differences between groups. The multivariate analysis showed that the clinical variables significantly associated with the outcome “death” were age > 34 years, MI and BI, length of stay, time since injury > 5 h, ISS > 17, and presence of associated injuries. These findings are hard to compare with previous reports from dissimilar settings examining factors related to mortality in patients with penetrating abdominal war wounds*.* For example, Iflazoglu et al. [34] found that in 120 patients with penetrating abdominal firearm injuries, factors significantly associated with death were number of blood transfusions, ISS and penetrating abdominal trauma index scores, number of injured organs, and presence of shock, but the patients were treated in a referral civilian hospital during the civil war in southern Turkey. In a group of 325 patients treated in a civilian hospital in Damascus for penetrating abdominal trauma from terror-related attacks from 2012 to 2013, Arafat et al. [25] reported that factors affecting mortality were duration of ICU stay, number of blood transfusions > 3 units, and PATI score > 25. The only paper in a similar setting, a review from Red Cross Field hospitals by Leppäniemi [10], reports in abdominal war wounds a mortality ranging from 8 to 25%, with no difference between patients who presented before or 12 h after injury and no data analyzing other possible factors affecting mortality.

## Conclusions

Our findings show that epidemiology and patterns of injury in patients with penetrating abdominal war injuries in a limited resources civilian hospital in Afghanistan differ from those reported in a typical military setting. Study population is mainly civilian with a significant number of women and patients under 14 years. BI are significantly more frequent than blast injuries, and gastrointestinal injuries were more common than injuries to solid organs. In this austere setting, surgeons need to acquire a wide range of skills from multiple surgical specialties. Outcome depends crucially on the major risk factors for mortality: bullet injury, age > 34, length of hospital stay, time since injury > 5 h, ISS > 17, and associated injuries. These findings might serve as a guide to trauma and general surgeons when treating penetrating abdominal war wounds to achieve better care and outcome.

## Data Availability

The datasets generated during and/or analyzed during the current study are available from the corresponding author on reasonable request.

## Abbreviations

ARDS: Adult respiratory distress syndrome
ATLS: Advanced trauma and life support
BI: Bullet injury
DCL: Damage control laparotomy
FAP: First aid post
GSW: Gunshot wounds
ICRC: International Committee of the Red Cross
ICU: Intensive care unit
ISS: Injury severity score
IVC: Inferior vena cava
MI: Mine injury
NGO: Non-governmental organization
OEF: Operation Enduring Freedom
OIF: Operation Iraqi Freedom
QID: Quarter in die
SI: Shell injury
SW: Stab wound
TID: *Ter in die*
